# Full-Arch Rehabilitation With Mucosa-Supported Prostheses Utilizing a Digital Workflow: A Case Report

**DOI:** 10.7759/cureus.64941

**Published:** 2024-07-19

**Authors:** Edgar Garcia, Ting Wei Tung, Stephanie Jaramillo, Alberto Gutierrez, Julio Alvear, Maurício Tinajero

**Affiliations:** 1 Prosthodontics, Universidad de Especialidades Espiritu Santo, Guayaquil, ECU; 2 Prosthodontics, University of Specialties Holy Spirit, Guayaquil, ECU; 3 Dentistry, Private Practice, Miranda, VEN; 4 Dentistry, Private Practice, Guayaquil, ECU; 5 Periodontology, Universidad de Especialidades Espiritu Santo, Guayaquil, ECU

**Keywords:** fulll-arch edentulism, complete dentures, mucosa-supported prostheses, oral rehabilitation, cad-cam

## Abstract

The advent of digital workflows has revolutionized oral rehabilitation, offering excellent prosthesis and restoration adaptation while reducing work time. This case report aims to describe a full-arch rehabilitation protocol using mucosa-supported prostheses through a digital workflow. The technique begins with scanning the upper jaw and taking an impression of the lower jaw, followed by scanning to create a digital cast. Next, border molding and the final impression of both arches are performed using a closed-mouth technique, during which the patient is guided to perform lateral and protrusive movements. Subsequently, intraoral scanning of the occlusion and both impressions is conducted, leading to the design of the final dentures with the established occlusion. Finally, the dentures are printed in Formlabs resin specifically designed for dentures. The digital workflow facilitates the manufacturing of mucosa-supported full-arch prostheses effectively. This method allows for the adjustment of the vertical dimension of occlusion, ensures excellent adaptation of the prosthesis to the soft tissues, and provides aesthetic satisfaction for the patient. Additionally, it reduces the number of sessions required to install the definitive prosthesis.

## Introduction

Total edentulism remains a significant clinical condition affecting a considerable portion of the global population (between 20% and 40%) [1,2]. The complete loss of teeth substantially impacts individuals’ quality of life, as it is associated with reduced chewing efficiency [3], social integration challenges due to aesthetic and phonetic issues [4], premature facial aging [5], and psychological problems stemming from decreased self-esteem [6].

Traditionally, mucosa-supported prostheses have been employed to treat total edentulism, achieving high success and satisfaction rates among patients [7,8]. However, several factors complicate this type of rehabilitation, including the number of sessions required to fabricate the definitive prosthesis, patient adaptation to the prosthesis, particularly in the mandible due to alveolar mucosa trauma, and the adjustment to the new occlusion. This is especially challenging for patients with edentulism or occlusal wear, which leads to a reduction in the vertical dimension of occlusion and inadequate adaptation of the chewing muscles and temporomandibular joint to the edentulous condition [7,9,10].

The advent of digital workflows has revolutionized dentistry, particularly in oral rehabilitation [11,12]. This technology involves scanning intraoral structures to create digital models, which are then used to manufacture prostheses or restorations via 3D printing [10,13]. Implant-supported and tooth-supported prostheses produced using this technology demonstrate high adaptation accuracy and good occlusal adjustment [12,13]. Additionally, digital workflows reduce the need for traditional impression materials and decrease the number of sessions required to complete definitive rehabilitation [12,13]. The use of digital workflow in mucous-supported full-arch rehabilitation has not been frequently addressed [10,11]. This fact is a gap in knowledge since most patients need this type of rehabilitation, either permanently or as a way of preparing for full-arch rehabilitation supported by dental implants. The clinical application of the digital workflow may reduce the time and costs of mucous-supported full-arch rehabilitation. The objective of this series of cases is to describe a complete arch rehabilitation protocol with mucous-supported prostheses through digital workflow.

## Case presentation

An 83-year-old female patient presented to the clinic with the chief complaint that her current dentures lacked retention. Her medical history was noncontributory and presented no contraindications for proceeding with dental treatment. An intraoral examination revealed an edentulous maxillary arch with a small residual ridge, minimal tuberosities, and no existing pathology affecting the prognosis. The mandibular arch displayed minimal retromolar pads and a significantly resorbed residual ridge.

**Figure 1:**
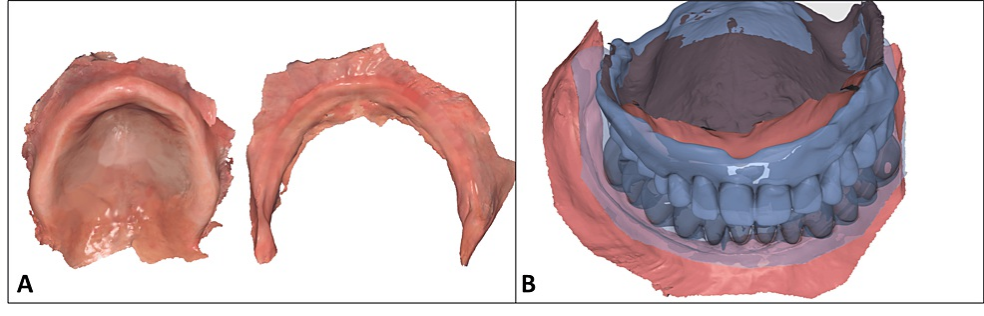
(A) Intraoral scans of edentulous maxillary arches. (B) Intraoral scans matched with the preliminary vertical dimension of occlusion (VDO) registration from previous dentures.

During the first appointment, both the upper and lower jaws were scanned using an intraoral scanner (Medit i500; Medit, Seoul, South Korea) to create a digital cast (Figure 1A). Additionally, light-body silicone was used to reline the patient’s current dentures, ensuring the meshes were aligned and could be used as a reference for the vertical dimension of occlusion (Figure 1B). The scans were exported to Exocad for prototype design.

**Figure 2:**
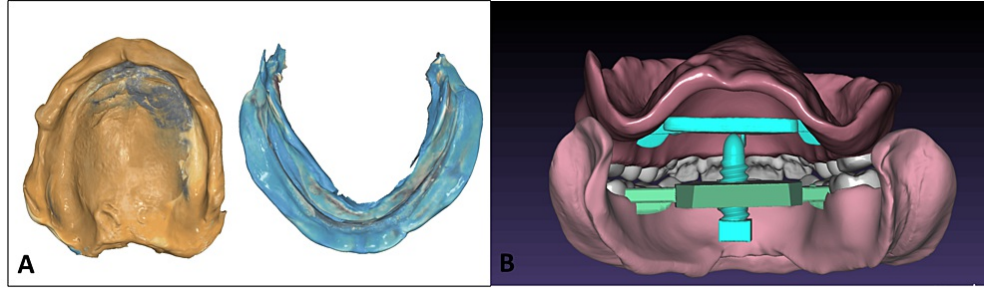
(A) Scans of wash impressions. (B) Prototype design with attached gothic arch files.

In the second appointment, border molding and the final impressions of both arches were performed using a closed-mouth technique with heavy-body vinyl polysiloxane (Heavy Body VPS; PlastCare USA, Los Angeles, CA) for the border molding and light-body vinyl polysiloxane (Light Body VPS; PlastCare USA) for the wash impression. These impressions were then scanned using an intraoral scanner (Medit i500) (Figure 2A) and exported to the design software (Exocad). Using Exocad, new prototypes were designed for the gothic arch tracing to be performed in the third appointment. For the upper prototype, a flat structure was integrated into the palate, while the lower prototype was designed without anatomical teeth but equipped with a screw to contact the upper jaw for tracing the gothic arch (Figure 2B).

**Figure 3:**
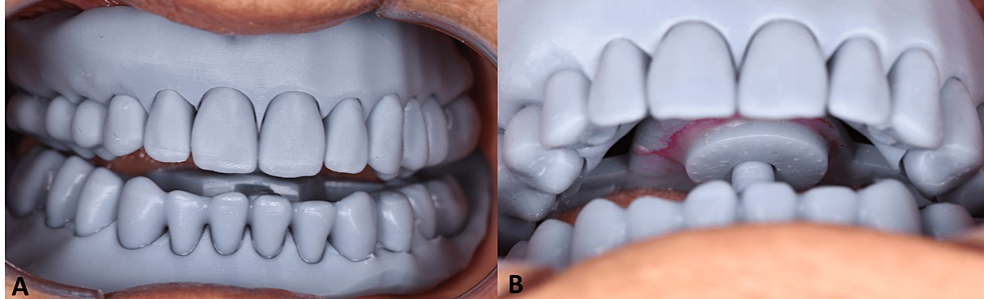
(A) Intraoral view of the printed prototype with the gothic arch tracer. (B) Screw of the gothic arch tracer secured for stable occlusal registration.

**Figure 4:**
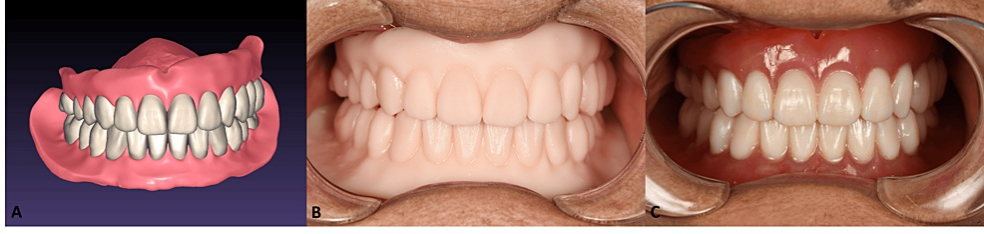
(A) Final design of the complete dentures in the design software. (B) Intraoral view of the denture prototypes. (C) Final printed complete dentures in the mouth.

During the third appointment, the patient was guided to perform lateral and protrusive movements to mark lines on the surface, identifying the tentative centric relation (Figure 3A). A printed circle was placed on the screw’s first portion to stabilize the mandibular position (Figure 3B). Subsequently, the occlusion and both impressions were scanned intraorally, and the final dentures were designed in Exocad with the established occlusion (Figure 4A). Finally, denture prototypes were printed in prototype resin (Solflex prov material; W2P Engineering, Vienna, Austria) to verify the size, shape of teeth, and occlusal record (Figure 4B). The final dentures were then printed in Formlabs resin specifically for dentures, with the base and teeth fabricated accordingly (Figure 4C).

The new dentures were delivered and adjusted as needed. The patient reported improved function and retention, expressing satisfaction with the overall fit and aesthetics of the new prostheses. She was instructed to return for routine follow-up appointments to ensure continued comfort and function.

## Discussion

The application of digital workflows for the manufacture of full-arch dentures has been less investigated, despite the significant demands and complexities associated with this technique. These complexities arise from the difficulties patients face in adapting to mucosa-supported prostheses and establishing the vertical dimension of occlusion [11,12]. These factors are critical as they affect the patient’s adaptation to prostheses. The proposed technique aims to enhance the adaptation of soft tissues to the prosthetic base by scanning oral structures and incorporating gothic arch tracing within the digital workflow.

Oral scanning is influenced by several factors, including the type of scanner, the patient’s edentulism type, and the operator’s experience. Edentulous areas are particularly challenging to scan due to the software’s difficulty in identifying references that ensure image continuity [14]. This challenge is more pronounced in lower edentulous arches, where significant alveolar process atrophy and high tongue floor insertion further complicate scanning [14]. Therefore, meticulous attention was given to ensuring high accuracy in regions difficult to scan, such as employing conventional molding of the lower arch followed by scanning this model for subsequent software assembly. Operator training and experience are crucial for the technique’s success, as experienced operators produce higher-quality models in less time and with smaller file sizes [15,16].

An essential aspect of the technique is the application of gothic arch tracing, which objectively and simply retains the centric relation and vertical dimension of occlusion data, thus aiding in correcting any musculoskeletal disorders, a significant consideration during edentulism treatment [17]. The gothic arch also tests the harmony of mandibular movements, which is critical as muscle pain is a common complaint during the adaptation phase to mucosa-supported prostheses [18].

Another key factor is the use of printed resins for the complete mucosa-supported dentures. These resins must possess adequate resistance to perform their function effectively. The prosthetic base, made with printed resins, has demonstrated good fracture resistance, especially when the thickness exceeds 1.5 mm [19]. Additionally, the resins used for the teeth exhibit wear resistance comparable to conventional denture teeth [20].

Finally, an advantage of using digital workflows for manufacturing full-arch prostheses is the reduction in the number of sessions and time needed to install the prostheses. While traditional materials for complete dentures may have lower costs, the overall reduction in the number of sessions and residual production makes the digital workflow for manufacturing full-arch mucosa-supported prostheses more cost-effective [11]. This cost-effectiveness is particularly beneficial for populations of lower socioeconomic status, who are more likely to require such prostheses.

## Conclusions

The digital workflow enables the efficient manufacture of full-arch mucosa-supported prostheses. This method allows for precise adjustment of the vertical dimension of occlusion, promotes excellent adaptation of the prosthesis to soft tissues, and ensures aesthetic satisfaction for the patient.
